# The concept of foam as a drug carrier for intraperitoneal chemotherapy, feasibility, cytotoxicity and characteristics

**DOI:** 10.1038/s41598-020-67236-7

**Published:** 2020-06-25

**Authors:** Justyna Schubert, Tanja Khosrawipour, Sören Reinhard, Mohamed Arafkas, Alice Martino, Jacek Bania, Marek Pieczka, Alessio Pigazzi, Veria Khosrawipour

**Affiliations:** 1 Department of Food Hygiene and Consumer Health Protection, Wroclaw University of Environmental and Life Sciences, 50-375 Wroclaw, Poland; 2 Division of Colorectal Surgery, Department of Surgery, University of California Irvine (UCI), 92868 Orange, USA; 3 Department of Surgery (A), Heinrich-Heine-University and University Hospital Duesseldorf, 40225 Duesseldorf, Germany; 4 Department of Bioengineering, University of California Berkeley (UC-Berkeley), 94704 Berkeley, USA; 5 Department of Plastic Surgery, Ortho-Klinik Dortmund, D-44263 Dortmund, Germany; 6 Department of Biochemistry and Molecular Biology, Faculty of Veterinary Sciences, Wroclaw University of Environmental and Life Sciences, 50-375 Wroclaw, Poland

**Keywords:** Pharmacokinetics, Surgical oncology, Drug delivery, Preclinical research, Translational research

## Abstract

For decades, intraperitoneal chemotherapy (IPC) was delivered into the abdominal cavity as a liquid solution. This preliminary study aims to evaluate foam as a potential new drug carrier for IPC delivery. Foam-based intraperitoneal chemotherapy (FBIC) was produced with taurolidine, hydrogen peroxide, human serum, potassium iodide and doxorubicin/ oxaliplatin for both *ex vivo* and *in vitro* experiments. Analysis of FBIC efficacy included evaluation of cytotoxicity, tissue penetration, foam stability, temperature changes and total foam volume per time evaluation. FBIC showed penetration rates of about 275 ± 87 µm and higher cytotoxicity compared to controls and to conventional liquid IPC (*p* < 0.005). The volume of the generated foam was approximately 50-times higher than the initial liquid solution and temporarily stable. Foam core temperature was measured and increased to 47 °C after 9 min. Foam ingredients (total protein content) were evenly distributed within different locations. Our preliminary results are quite encouraging and indicate that FBIC is a feasible approach. However, in order to discuss a possible superior effect over conventional liquid or aerosolized chemo applications, further studies are required to investigate pharmacologic, pharmacodynamic and physical properties of FBIC.

## Introduction

Even after decades of clinical and experimental research, the treatment of Peritoneal metastasis (PM) still remains challenging with poor prognosis and median survival rates of only a few months^1^. In a highly selective group of patients with isolated peritoneal cancer, cytoreductive surgery (CRS) combined with hyperthermic intraperitoneal chemotherapy (HIPEC) offers a curative approach to PM^2,3^.

For the majority of PM patients who do not qualify for CRS and HIPEC, treatment options are mostly limited to systemic intravenous chemotherapies (IVC) or intraperitoneal chemotherapies (IPC). However, IVC was demonstrated to exert only a limited effect on PM^4,5^. While IPC is considered to improve local drug availability compared to intravenous delivery, it also displays major technical and prognostic limitations such as the risk of local complications caused by IPC devices, inhomogeneous drug distribution and limited drug penetration depth into the tissue^6,7^. These limitations have also been demonstrated for a more recent IPC application via aerosol formation, called pressurized intraperitoneal aerosol chemotherapy (PIPAC)^8–11^. However, as opposed to liquid and aerosol applications, foam has not been widely studied as a potential drug carrier in IPC. This is quite astonishing, as foam possesses unique features which may result in superior drug effects of foam-based intraperitoneal chemotherapy (FBIC) over current IPC applications.

To evaluate whether FBIC is a feasible tool for IPC, we investigated its cytotoxicity *in vitro*, analyzed its structural stability and tested its characteristics in an *ex vivo* model mimicking the abdominal cavity. The two main substances used in our model, taurolidine and hydrogen peroxide, both display high antitumoral activity, which has recently been the subject of oncologic research. Taurolidine has shown to be highly cytotoxic in colon cancer, exhibiting similar cytotoxic levels as oxaliplatin^12^. Hydrogen peroxide has also demonstrated a wide range of specific antitumoral activity. Both endogenously produced and exogenously added hydrogen peroxide display an antitumor effect^13–15^. Unfortunately, the delivery of hydrogen peroxide into solid tumors is much more challenging than its common use as a surface applicant in the treatment of skin cancer^16^. Nevertheless, hydrogen peroxide has recently been tested in the treatment of solid tumors, among other substances^17,18^. Although its various antitumoral effects are increasingly understood and appreciated^19^, the implementation of a potentially widely applicable hydrogen peroxide solution for oncologic purposes remains challenging.

## Methods

### Foam-based intraperitoneal chemotherapy (FBIC)

The ratio of foam ingredients were experimentally determined. To create the FBIC solution of taurolidine (Taurolin® Ringer 0.5%, Berlin-Chemie AG, Berlin, Germany), hydrogen peroxide (30% hydrogen peroxide solution, Chempur, Piekary Śląskie, Poland), human serum (from human male AB plasma, Sigma-Aldrich; Merck KgaA, Darmstadt, Germany) and potassium iodide (Sigma-Aldrich; Merck KgaA) was used. The initial liquid solution consisted of 0.045% taurolidine, 22.8% hydrogen peroxide, 12.5% human serum and 12 mM potassium iodide. Additionally, doxorubicin (doxorubicin hydrochloride purchased from PFS®, 2 mg/ml, Pfizer, Sandwich, United Kingdom) and oxaliplatin (Medoxa, Medac GmbH, Wedel, Germany) were added in both *ex vivo* and *in vitro* models, respectively. The applied dosage of doxorubicin was chosen based on dosages used in PIPAC, e.g. 3 mg of doxorubicin was used to create foam covering a 4-liter cavity^11,20,21^. Oxaliplatin was applied at a total concentration of 26.8 µg/ml.

### *Ex vivo* model

**Figure 1:**
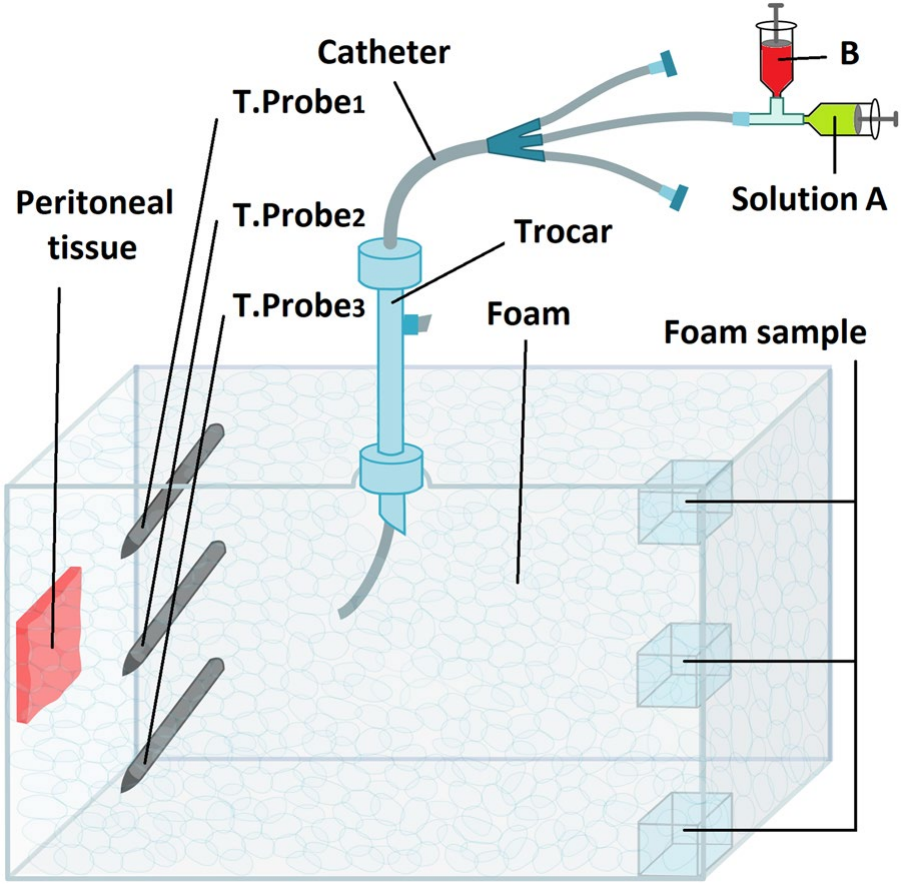
*Ex vivo* experiment on fresh swine peritoneum for FBIC investigation. Evaluation of foam stability, temperature (T.probes 1–3) and gravitational effects. A central venous catheter (CVC) was placed in the center of the top in a 5 mm trocar and used to apply the foam by injecting a previously mixed starting solution.

The experiments were performed in a standard *ex vivo* model on commercially available tissue samples, therefore no approval of the Institutional Review Board and no consent of the Local Board on Animal Care was required. The *ex vivo* model has been well established and previously described in many studies^22,23^. A commercially available hermetic plastic box with a total volume of 4 liters was used, mimicking the abdominal cavity. The plastic box was closed during each individual procedure. On the cover of the plastic box, a 5 mm trocar (Kii®Balloon Blunt Tip System, Applied Medical, Rancho Santa Margarita, CA, USA) was placed. Using one trocar, a medical intravenous catheter was introduced. The catheter was used to apply the foam by injecting a previously mixed starting solution (containing taurolidine, hydrogen peroxide, human serum and doxorubicin) and potassium iodide to induce foam formation. Three fresh tissue specimen of peritoneum (German landrace pigs), each measuring 3.0 ×3.0 ×0.5 cm, were placed at the side wall (Fig. 1). Drug-exposure time to FBIC was 30 min.

### Evaluation of foam stability, measurement of temperature and gravitational effects

30 ml of the initial FBIC solution was introduced in a measuring jug to produce 4 liters of foam. FBIC volume was measured after initiating foam creation by addition of potassium iodide, which served as a catalyst. Central and peripheral temperatures were measured via a temperature probe at different time points of the foam formulation process (Fig. 1). Time points for temperature measurements were equivalent to 0, 25, 50, 75 and 100% of the estimated maximal total volume of the foam produced. When the foam reached its maximum expansion, volume samples (0.25 ml) were taken from the bottom, middle and top of the foam. To evaluate possible gravitational effects in the foam composition, the protein fraction of the human serum was used as an indicator of possible concentration imbalances. Using the Bradford method (Sigma-Aldrich), the total content of human serum protein was determined to detect possible concentration differences between different areas of the foam.

### Microscopic analysis

After treatments, all tissue samples were rinsed with sterile NaCl 0.9% solution to eliminate superficial cytostatic agents and immediately frozen in liquid nitrogen. Cryosections (7 µm) were prepared from different areas of the specimen. Sections were mounted with VectaShield containing 1.5 µg/ml 4’,6-diamidino-2-phenylindole (ProLong® Gold Antifade Reagent with DAPI, Thermo Fisher Scientific) to stain nuclei. Penetration depth of doxorubicin was measured using a Nikon Eclipse 80i fluorescence microscope (Nikon Instruments Europe B.V. Amsterdam, Netherlands). The distance between the peritoneal surface and the innermost positive staining for doxorubicin accumulation was reported in micrometers.

### Cytotoxicity of FBIC on colon cancer cells (HT-29) *in vitro*

The human colorectal cancer cell line HT-29 was obtained from CLS (Cell Lines Service GmbH, Eppelheim, Germany) and cultured in DMEM (Dulbecco’s modified Eagle’s medium; Sigma-Aldrich) supplemented with 10% heat-inactivated fetal calf serum (Invitrogen, Warsaw, Poland), 2 mmol/l glutamine, 100 IU/ml penicillin, and 100 μg/mL streptomycin (Sigma-Aldrich) at 36 °C in a humidified 5% CO_2_/air atmosphere. HT-29 cells were seeded at a density of 1.4 ×10^5^ cells per well in 24-well plates (TC Plate 24 Well, Standard, F, Sarstedt AG & Co. KG, Germany) and incubated for 48 hours at 36 °C with 5% CO_2_.

Next, to investigate the effect of FBIC treatment on tumor cell cytotoxicity, the medium was aspirated from each well. The attached cells were treated with four different solutions:

- 0.5 ml of regular cell medium;
- 0.5 ml of cell medium plus oxaliplatin
- 1 ml of foam;
- 1 ml of foam plus oxaliplatin;

The exposure time was 1 hour at 36°C with 5% CO_2_. After this period, the content of the wells was aspirated, and 0.5 ml of fresh medium was added. Cells were incubated for 48 hours under the same conditions and an MTS proliferation assay was performed.

### MTS test

An MTS (3-(4,5-dimethylthiazol-2-yl)-5-(3-carboxymethoxyphenyl)-2-(4-sulfophenyl)-2H-tetrazolium) assay was performed according to the manufacturer’s instruction (Promega GmbH, Mannheim, Germany) with modifications. Briefly, the medium was removed from each well and replaced by 0.3 ml of fresh DMEM. 60 µl of CellTiter 96® AQueous One Solution Reagent was added to each well and absorbance was measured at 490 nm on a microplate reader (Tecan, Basel, Switzerland) after 1 hour of incubation at 37°C and the percentage of proliferation was determined for all groups.

### Statistical analyses

Experiments were independently performed in triplicates. The statistical analyses were performed with GraphPad Prism (GraphPad Software Inc., version 8.0.2 (263)). The Kruskal-Wallis One Way Analysis of Variance on Ranks was used to compare independent groups. Descriptive statistics included mean, median and percentiles. Probability *(p)* values were considered as follows: *p < 0.05 and **p < 0.005, and #*p* > 0.05, with *p*-value <0.05 considered to be statistically significant.

### Ethical approval and regulations

The human cancer cells were commercially acquired. Thus, according to the Minister of Science and Higher Education Poland of 5 May 2015 on the National Ethics Committee for Human and Animal Experiments and local ethical commissions no ethical approval was required for this study. However a request was made and protocol was approved (411 A/2019) by the Ethics Committee of Wroclaw University of Environmental and Life Sciences, 50–375 Wroclaw, Poland.

Part of the experiments were performed on commercially available animal tissue samples. All methods were carried out in accordance with relevant guidelines and regulations which apply according to polish law. An Approval of the Local Board on Animal Care was obtained (Zapytanie 8/8/2019) according to the Polish law.

## Results

### *Foam stability, measurement of temperature and gravitational effects*

**Figure 2:**
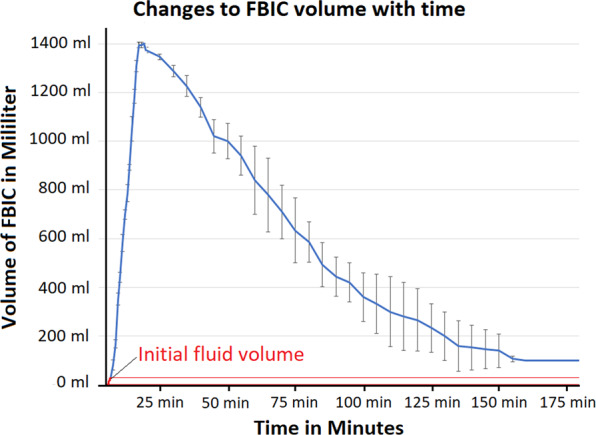
Changes of foam volume with time (blue line) demonstrated with standard deviation at each time point. Initial fluid volume from which foam was created is demonstrated by a separate (red) line.

**Figure 3:**
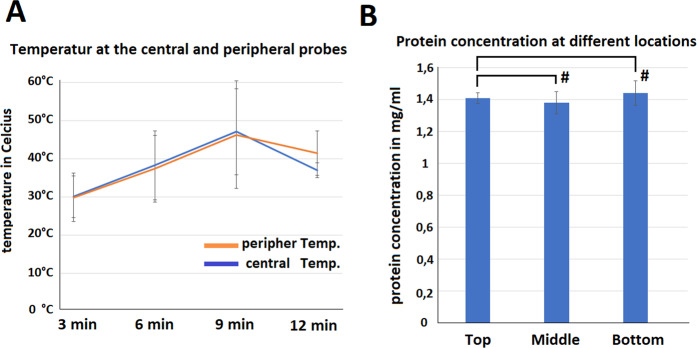
(**A**) Core and peripheral temperatures of the foam at specific time points. Time points were chosen according to 0, 25, 50, 75 and 100% of the estimated maximal total volume of the foam. (**B)** Total protein content of human serum dissolved in foam at its bottom, middle and upper parts.

The formation of a temporarily stable foam was possible. The foam reached its maximal volume after 12 minutes, after which volume size constantly decreased (Fig. 2). The volume of the foam produced was approximately 50 times greater than the initial liquid solution. The foam was stable for 150 minutes. The foam volume decreased by 50% after 74 minutes and 75% after 113 minutes. The temperature of central and peripheral parts of the formulated foam was measured at time points equivalent to 0, 25, 50, 75 and 100% of the estimated maximum total volume of the foam. Core (temperature probe 2) and peripheral temperatures (temperature probes 1 and 3) of the foam rose up to 47 and 46 °C, respectively, after 9 min (Fig. 3A). Foam total protein content was evenly distributed on the bottom, in the middle and at the top of the box. No significant differences in the concentration of the protein could be detected (*p* > 0.05, Fig. 3B).

### Microscopic analysis of doxorubicin depth penetration in the *ex vivo* model

**Figure 4:**
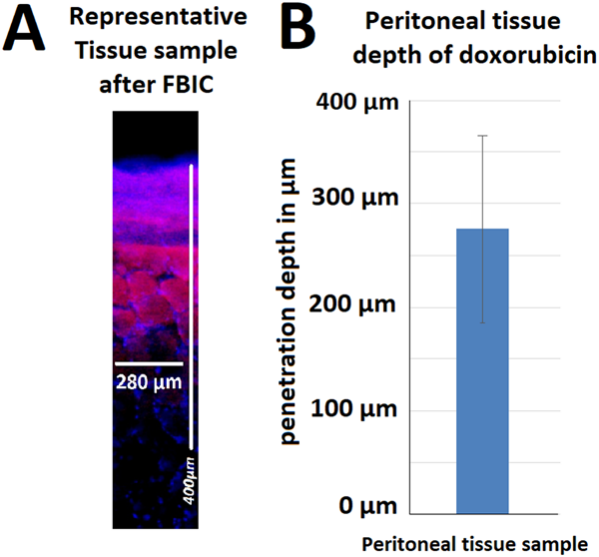
(**A**) Microscopic analysis of the penetration depth of a representative sample of doxorubicin (red) into fresh peritoneal tissue samples of German landrace pigs. Nuclei (blue) were stained with 4’,6-diamidino-2-phenylindole (DAPI). (**B)** Distribution of penetration depths is presented in µm with mean and standard deviation.

All samples had contact with the FBIC in the established *ex vivo* model. A minimal amount of foam remained constantly on the sample surface at all times. A partial discoloration of the superficial peritoneal layer could be observed after removing the foam layer. The mean depth of doxorubicin penetration detected via fluorescence microscopy was found to be 275 ± 87 µm (Fig. 4). Measured penetration depths are higher at peripheral locations in FBIC than in tissue samples treated with liquids and pressurized aerosol^8,24^. The volume of the foam produced decreased with time, as the foam structure destabilized.

### Cytotoxicity of FBIC on colon cancer cells (HT-29) in vitro

**Figure 5:**
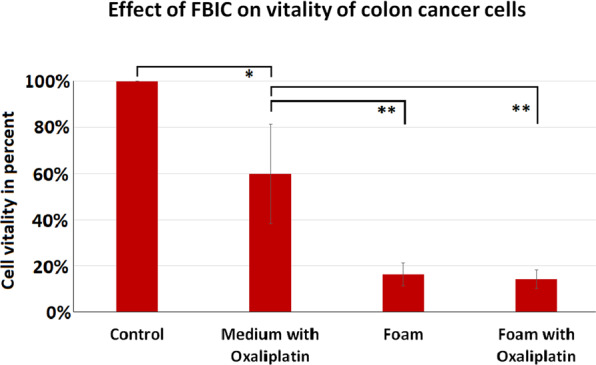
*In vitro* cytotoxic effect of foam on colon cancer cells (HT-29). Vitality of colon cancer cells presented in comparison to untreated control samples (A, B) in % with and without addition of oxaliplatin (*p < 0.05, **p < 0.005, ^#^*p* > 0.05).

FBIC showed significant cytotoxicity compared to untreated controls in an *in vitro* model (*p* < 0.05, Fig. 5). Interestingly, FBIC seems to have even higher cytotoxicity than medium with oxaliplatin (*p* < 0.005). This effect remains unchanged even if oxaliplatin is added to FBIC. No additional cytotoxicity is observed in that case.

## Discussion

Intraperitoneal administration of anticancer drug solutions is an established method of treating PM, as highly concentrated drug particles are put into contact with tumor nodules in the peritoneal cavity. However, limitations such as inhomogeneous drug distribution and limited penetration into the peritoneal tissue have been described with both liquid as well as aerosol-based IPC^6,8^, even when applied with new, improved techniques^20,24,25^. Additionally, novel substances delivered intraperitoneally have displayed limitations in drug tissue penetration^26^ and modified technical applications have been suggested to improve IPC^27^.

**Figure 6:**
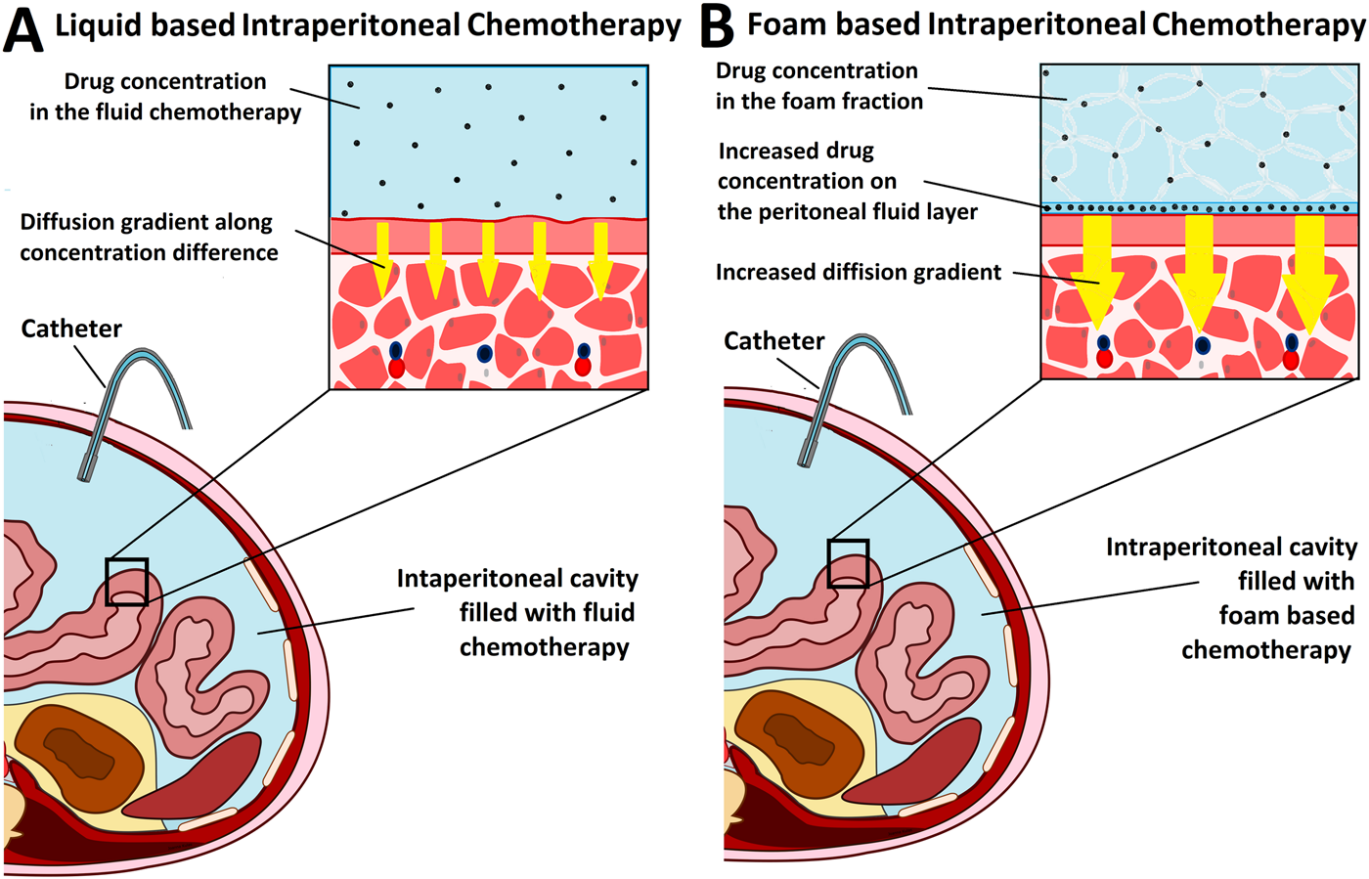
Schematic representation of liquid vs. foam based chemotherapy in an abdominal model. (**A**) Diffusion gradient is based on drug concentration of the chemotherapeutic solution in liquid based chemotherapy. (**B**) While the concentration gradient of the foam fraction is the same as in liquid based chemotherapy, the concentration increases during foam degradation on the peritoneal surface. The thin fluid layer has direct contact to the underlying peritoneum, and the fluid film has high drug concentrations resulting in a much higher diffusion gradient.

Foam displays some unique characteristics which, to the best of our knowledge, have not been tested as a drug carrier for intraperitoneal chemo applications. Foam-based IPC could be a technically feasible option for treatment of PM, using doxorubicin and oxaliplatin as in this study or other chemotherapeutic agents. Penetration depths of chemotherapy are higher compared to liquid or aerosol-based IPC, especially when compared to penetration levels at more peripheral locations. This study also demonstrates that foam volume decreases with time. Slow degradation allows for extended drug contact time with the peritoneal tissue and a higher drug diffusion gradient (Fig. 6). This is an interesting feature, since length of contact of a chemotherapeutic drug with the peritoneal tissue enhances drug availability and efficiency.

Foam has many advantages over both aerosol and liquid applications. It expands differently than liquids and gas and displays a higher drug-carrying capacity than gas. Thus, even a low total drug dosage can create high drug concentrations as more than 95% of the actual volume is air. Aerosol chemotherapy has been shown to achieve much higher drug concentrations than regular liquid solutions. However, this increase in drug concentration is not without consequence, as aerosol chemotherapy displays increased inhomogeneity compared to liquid applications.

The unique characteristics of foam might significantly improve the response of PM to IPC. In our study, foam containing hydrogen peroxide and taurolidine has demonstrated cytotoxic properties which may be sufficient for the treatment of PM without the need for additional chemotherapeutic agents. However, further studies are needed to evaluate the clinical applications of taurolidine and hydrogen peroxide foam in the treatment of PM.

Our data indicate that foam might be a possible carrier for IPC and could offer increased drug penetration and more homogenous drug distribution than conventional liquids and pressurized aerosol. However, further research is required to assess its potential in IPC application. To the best of our knowledge, no clinical experience for foam-based applications in IPC has been previously collected or published in peer-reviewed literature. While this study presents preliminary data, it gives important insight into the potential of FBIC to improve PM treatment and encourages further studies to evaluate FBIC’s full efficacy and biodistribution.

## Data Availability

The datasets used and/or analyzed during the current study are available from the corresponding author upon reasonable request.
